# Comparison of Hospital Readmission After Total Hip and Total Knee Arthroplasty vs Spinal Surgery After Implementation of the Hospital Readmissions Reduction Program

**DOI:** 10.1001/jamanetworkopen.2019.4634

**Published:** 2019-05-31

**Authors:** Ashwin Ramaswamy, Maya Marchese, Alexander P. Cole, Sabrina Harmouch, David Friedlander, Joel S. Weissman, Stuart R. Lipsitz, Adil H. Haider, Adam S. Kibel, Andrew J. Schoenfeld, Quoc-Dien Trinh

**Affiliations:** 1 Icahn School of Medicine at Mount Sinai, New York, New York; 2 Division of Urological Surgery, Brigham and Women’s Hospital, Harvard Medical School, Boston, Massachusetts; 3 Center for Surgery and Public Health, Brigham and Women’s Hospital, Harvard Medical School, Boston, Massachusetts; 4 Division of Orthopedic Surgery, Brigham and Women’s Hospital, Harvard Medical School, Boston, Massachusetts

## Abstract

**Importance:**

The Hospital Readmissions Reduction Program (HRRP) was recently expanded to penalize excessive readmissions after total hip arthroplasty (THA) and total knee arthroplasty (TKA). These are the first surgical procedures to be included in the HRRP.

**Objective:**

To determine whether the HRRP was associated with a greater decrease in readmissions after targeted procedures (THA and TKA) compared with similar nontargeted procedures (lumbar spine fusion and laminectomy).

**Design, Setting, and Participants:**

A retrospective cohort study was conducted of patients 50 years or older among all payers in the Nationwide Readmissions Database who underwent THA, TKA, lumbar spine fusion, or laminectomy between January 1, 2010, and September 30, 2015. Multivariable logistic regression and interrupted time-series models were used to calculate and compare 30-day readmission trends in 3 periods associated with the HRRP: preimplementation (January 2010-September 2012), implementation (October 2012-September 2014), and penalty (October 2014-September 2015). Statistical analysis was performed from January 1, 2010, to September 30, 2015.

**Exposures:**

Announcement and implementation of the HRRP.

**Main Outcomes and Measures:**

Readmission within 30 days after hospitalization for THA, TKA, lumbar spine fusion, or laminectomy surgery.

**Results:**

The study included 6 687 077 (58.3% women and 41.7% men; mean age, 66.7 years; 95% CI, 66.7-66.8 years) weighted hospitalizations for THA, TKA, lumbar spine fusion, and laminectomy surgery: 4 765 466 hospitalizations for targeted conditions and 1 921 611 for nontargeted conditions. After passage of the Patient Protection and Affordable Care Act, the risk-adjusted rates of readmission after all procedures decreased in a similar fashion. Implementation of the HRRP was associated with a 0.018% per month decrease in the rate of readmission (95% CI, −0.025% to −0.010%) after targeted procedures, which was not observed after nontargeted procedures (slope per month, −0.003%; 95% CI, −0.016% to 0.010%). Penalties were not associated with a greater decrease in readmission for either targeted or nontargeted procedures.

**Conclusions and Relevance:**

These results appear to be consistent with hospitals responding to the future possibility of penalties by reducing readmissions after surgical procedures targeted by the HRRP.

## Key Points

**Question:**

Is the US Hospital Readmissions Reduction Program associated with a greater decrease in unplanned readmissions after targeted surgical procedures when compared with similar nontargeted procedures?

**Findings:**

In this nationwide, all-payer cohort study of 6 687 007 weighted index surgical admissions, implementation of the Hospital Readmissions Reduction Program was associated with a decrease of 0.018% per month in the risk-adjusted readmission rate after targeted procedures, while the readmission rate after nontargeted procedures remained constant, a difference that was statistically significant.

**Meaning:**

Readmission trends appear to be consistent with hospitals’ response to the possibility of Hospital Readmissions Reduction Program penalties after total hip arthroplasty and total knee arthroplasty.

## Teaser

This cohort study examines whether the US Hospital Readmissions Reduction Program was associated with a greater decrease in readmissions after targeted procedures (total hip arthroplasty and total knee arthroplasty) compared with similar nontargeted procedures (lumbar spine fusion and laminectomy).

## Introduction

Decreasing readmissions after hospitalization has become an established priority and a benchmark of health care quality in the United States. As a result, the Patient Protection and Affordable Care Act (ACA) established the Hospital Readmissions Reduction Program (HRRP) to start penalizing hospitals for excessive readmissions for select medical conditions under Medicare starting in 2012.^1^ In 2014, the HRRP was expanded to include surgery for the first time by penalizing excessive readmissions after total hip arthroplasty (THA) and total knee arthroplasty (TKA). Hospitals became familiar with planned inclusion of THA and TKA procedures in the HRRP in September 2012.^2^ Total hip arthroplasty and TKA were chosen because of their high prevalence, readmission rates, and overall expense to Medicare.^3,4^ The HRRP has been associated with reduced readmissions after hospitalization for targeted medical conditions.^5,6^

In this context, we sought to assess changes in 30-day risk-adjusted rates of readmission after targeted procedures (TKA and THA) vs similar nontargeted orthopedic procedures (lumbar spinal fusion and laminectomy) among patients 50 years or older between 2010 and 2015 using the Nationwide Readmissions Database (NRD), a prospectively maintained administrative database from the Healthcare Cost and Utilization Project (HCUP).^7^ To our knowledge, this study is the first to evaluate the association of the HRRP with surgical readmissions after penalty onset and with a newly available data set designed to study readmissions.^8,9,10^ Our hypothesis is that 30-day readmissions after targeted procedures would experience a more pronounced decline during the HRRP implementation and penalty periods when compared with readmissions after similar nontargeted procedures. If true, this finding would argue that the HRRP is associated with a greater decrease in readmissions after targeted surgical discharges.

## Methods

### Data Source

Data were derived from the January 1, 2010, to September 30, 2015, releases of the NRD.^7^ The NRD contains a nationally representative, stratified, clustered sample on hospital readmissions (including nonindex readmissions) for all payers and uninsured patients, representing 21 geographically distinct states accounting for nearly half of all US hospitalizations. It was constructed as a compilation of 85% of all discharges from the individual HCUP State Inpatient Databases and covers a weighted nationally representative sample of approximately 35 million individuals. The database contains deidentified patient linkage numbers that can track patients across hospitals, between hospitals, and within a state. This study was approved by the Brigham and Women’s Hospital Institutional Review Board, under a general study protocol for analyses using HCUP data and is fully compliant with the Strengthening the Reporting of Observational Studies in Epidemiology (STROBE) reporting guidelines for cohort studies.^11^ Patient consent was waived as data were deidentified.

### Study Population

We performed a retrospective cohort study of patients 50 years or older in the NRD between 2010 and 2015 undergoing 1 of 4 surgical procedures: THA, TKA, lumbar spine fusion, and laminectomy. A secondary analysis was performed evaluating the procedures of interest among Medicare patients. The NRD excludes patients whose index hospitalization was in December of any of the study years, as these patients’ 30-day readmission may be outside of the NRD database (ie, in January of the following year). Healthcare Cost and Utilization Project discharge weights were applied to obtain a sample representative of the overall US population.

We defined the study group as consisting of 2 joint procedures targeted by the HRRP (THA and TKA) and the control group as consisting of 2 other similar surgical procedures not currently targeted by the HRRP (lumbar spine fusion and laminectomy). Lumbar spine fusion and laminectomy were included in the control group because they are clinically similar to THA and TKA: these procedures are complex, invasive, mostly elective orthopedic procedures with high rates of postsurgical readmissions^12^ and are performed in similar patient populations. Total hip arthroplasty, TKA, and lumbar spine fusion all involve the use of an implant. Lumbar spine fusion and laminectomy are procedures with similar indications because they both are used in the treatment of spinal stenosis and degenerative spondylolisthesis.^13,14^ Furthermore, THA, TKA, lumbar spine fusion, and laminectomy together account for most adverse events in orthopedic surgery.^15^ Only patients 50 years or older were included because this is the population of interest in readmission reduction efforts.^16,17^ The NRD was queried using *International Classification of Diseases, Ninth Revision, Clinical Modification* (*ICD-9-CM*) procedure codes to identify patients with specific diagnoses who were undergoing these surgical procedures (eTable 1 in the Supplement).

We defined 3 separate periods corresponding to preimplementation (January 2010 to September 2012; 31 months), implementation (October 2012 to September 2014; 22 months), and penalty (October 2014 to September 2015; 11 months). The preimplementation period corresponds to the time prior to which hospitals became familiar with inclusion of THA and TKA into the HRRP. The implementation period corresponds to the time after hospitals became familiar with inclusion of measures for THA and TKA, but prior to application of HRRP penalties. The penalty period corresponds to the first year that hospitals experienced payment adjustments for excess readmissions for THA and TKA.

We applied the standard exclusion criteria of the HRRP for THA and TKA, excluding fractures of the pelvis or lower limbs, revision surgical procedures, and malignant neoplasms (eTable 2 in the Supplement). Current partial hip or knee arthroplasty were not excluded, as these were not distinguishable by *ICD-9-CM* codes. The NRD excludes patients whose index hospitalization was in December of any of the study years, as these patients’ 30-day readmission may be outside of the NRD database (ie, in January of the following year). The NRD does not allow tracking of patients or hospitals over study years, as patient linkage numbers and hospital identifiers do not track the same person or hospital in different years. In addition, nonresidents who underwent the index surgery in a state other than that of their primary residence were excluded; this was done because the NRD does not track individuals who are readmitted to a hospital in their home state. Patients listed in the NRD as having died during an index hospitalization were removed from the sample.^18^ Furthermore, October and November were excluded in 2015 as these months were coded with *International Statistical Classification of Diseases and Related Health Problems, Tenth Revision, Clinical Modification* procedure codes.

### Outcome

Our primary outcome was any patient readmission within 30 days of discharge after a hospitalization in which the procedures of interest were performed. Readmission is defined as the first inpatient hospitalization for any diagnosis after the index readmission. All readmissions that occurred within 30 days of discharge were included in this analysis with the exception of the exclusion criteria listed above. Cases in which a patient was transferred to another facility and planned readmissions were not defined as readmissions. Last, we excluded all hospitals that performed fewer than 25 of the procedures, per HCUP Data Use Agreement rules for reporting cell sizes that are fewer than 25 of observations.

### Covariates

Patient-level covariates included age, sex, Charlson Comorbidity Index,^19^ insurance status, and income (measured by zip code quartile). Hospital-level covariates included surgical procedure volume and hospital bed size. Surgical procedure volume was calculated by ranking hospitals by procedures performed and dividing facilities into 4 equal quartiles. Insurance status, surgical procedure volume quartile, bed size, and income were included as covariates because of the all-payer population of this study, previous inclusion in studies evaluating readmissions after surgical procedures,^20,21^ and to better control for variations in hospital size and patient income.

### Statistical Analysis

Statistical analysis was performed from January 1, 2010, to September 30, 2015. The association between the HRRP and readmissions was assessed with an interrupted time-series model estimating risk-adjusted rates of readmission for targeted and nontargeted procedures in each of the 3 study periods. Our primary comparisons were trends in readmission rates between targeted and nontargeted procedures within each study period. Our secondary comparisons were within-group comparisons of trends in readmission rates between successive periods; specifically, we compared period 1 vs 2 and period 2 vs 3 within groups (separately for targeted and nontargeted procedures).

Our objective was to investigate 3 questions. First, what were the trends in readmission rates for targeted and nontargeted procedures in each of the 3 periods? Second, did readmission trends within each study period differ significantly between targeted and nontargeted procedures (eg, targeted vs nontargeted procedures during HRRP implementation)? Third, did readmission trends differ significantly for each of the targeted and nontargeted procedures between study periods (eg, HRRP implementation vs penalty for targeted procedures)?

To assess these comparisons, we estimated the slope of readmission rates in each study period for targeted and nontargeted procedures. Specifically, within a single multivariable logistic regression model (eAppendix in the Supplement), we fit separate linear spline models for targeted and nontargeted procedures, allowing the trends to vary over time differently in each group in each of the 3 study periods. In this multivariable logistic regression model, the patient’s readmission status was the outcome variable (binary time), which was quantified in months, and patient-level and hospital-level variables were controlled for as covariates. To allow the slope within each group to change between periods, we set linear splines when 1 period finished and another started: at October 2012 (preimplementation to implementation) and October 2014 (implementation to penalty). The logistic model produces an odds ratio for the time trend of the odds of readmission for a 1-month increase within each of the 3 periods and 2 groups, giving 6 odds ratios for time trends. From the logistic model, we also calculated monthly risk-adjusted rates.

To assess whether readmission trends within each study period differed significantly in targeted vs nontargeted procedures, we tested for equal slopes between groups within a study period. To assess whether readmission trends differed in successive study periods within groups, we tested for equal slopes in period 1 vs 2 and period 2 vs 3 within a group. To account for the complex survey design, we adjusted for strata, cluster, and weight variables. Specifically, we defined strata as the NRD strata code, clusters as a unique combination of hospital and year, and survey weights per NRD’s survey weight variable.

All analyses were performed in Stata, version 14.0 (StataCorp), SAS, version 9.4 (SAS Institute Inc), and R, version 3.4.1 (R Project for Statistical Computing). All *P* values were from 2-sided tests and results were deemed statistically significant at *P* < .05.

## Results

### Index Admissions

Our weighted data set included 4 765 466 index admissions for targeted conditions (THA and TKA) and 1 921 611 index admissions for nontargeted conditions (lumbar spine fusion and laminectomy), among patients discharged from January 1, 2010, to September 30, 2015. During the study period, there were 1 580 760 THAs, 3 184 706 TKAs, 687 700 lumbar spine fusions, and 1 233 911 laminectomies. A larger proportion of both targeted and nontargeted procedures were observed in large hospitals with high case loads, in private nonprofit hospitals, and in hospitals in metropolitan areas (Table 1). Patients hospitalized for targeted procedures were more likely to be female and older than those hospitalized for nontargeted procedures. Between 2010 and 2015, there was an increase in age and comorbidities for patients admitted for both targeted and nontargeted procedures, with greater increases seen among nontargeted procedures; there was very little additional difference in the characteristics of patients admitted for targeted or nontargeted procedures or in the hospitals where they were treated.

**Table 1.** Weighted Annual Index Admissions and Patient and Hospital Characteristics

Variable	Targeted Conditions		Nontargeted Conditions	
	Jan 2010 to Nov 2010	Jan 2015 to Sep 2015	Jan 2010 to Nov 2010	Jan 2015 to Sep 2015
Patients, No.	748 871	736 260	324 462	272 295
Hospitals, No.	1354	1801	987	1202
Index admissions, No.				
Total hip arthroplasty	232 211	258 847	NA	NA
Total knee arthroplasty	516 660	477 413	NA	NA
Lumbar spine fusion	NA	NA	112 009	99 068
Laminectomy	NA	NA	212 453	173 227
30-d Readmission rate, %	4.8	4.1	7.4	7.3
Age, mean (95% CI), y	67.6 (67.6-67.7)	67.2 (67.2-67.3)	64.7 (64.7-64.8)	65.0 (65.0-65.1)
Age group, %				
50-59	22.4	22.1	35.0	32.3
61-70	35.7	38.7	33.7	35.5
71-80	29.7	29.0	23.4	25.3
>80	12.2	10.3	7.9	7.0
Sex, %				
Male	38.1	39.1	47.9	49.3
Female	61.9	60.9	52.1	50.7
Charlson Comorbidity Index, %^a^				
0	60.0	59.7	57.8	54.4
1	27.6	26.1	28.2	28.0
2	8.1	8.8	8.8	10.3
≥3	4.4	5.5	5.2	7.4
Volume quartile, %				
First	2.8	2.7	2.2	3.3
Second	10.0	9.7	10.6	11.3
Third	22.3	23.6	25.4	25.0
Fourth	64.9	63.9	61.8	60.4
Payer, %				
Medicare	58.5	59.2	51.3	55.2
Medicaid	2.3	3.7	3.0	5.0
Private insurance	35.3	33.7	37.0	32.4
Self-pay	0.3	0.3	0.7	0.6
Unknown	3.6	3.1	8.0	6.7
Income quartile, %				
First	22.5	21.5	24.1	24.1
Second	25.1	25.3	25.5	25.4
Third	25.3	28.3	25.5	27.3
Fourth	27.1	24.9	25.0	23.3
Elective, %				
Nonelective	8.2	5.3	15.9	15.0
Elective	91.8	94.7	84.1	85.1
Hospital urban-rural, %				
Large metropolitan (>1 million)	51.9	50.2	52.5	53.6
Small metropolitan (<1 million)	37.7	40.0	43.2	42.8
Micropolitan	8.6	7.6	4.1	3.2
Nonmetropolitan or micropolitan	1.7	2.2	0.2	0.1
Hospital owner, %				
Government	10.5	9.0	12.4	10.1
Private, nonprofit	75.2	77.1	70.9	73.5
Private, for-profit	14.2	14.0	16.8	16.4
Bed size, %				
Small	17.8	23.7	12.6	14.7
Medium	22.7	28.5	20.7	27.4
Large	59.5	47.9	66.7	58.0
Teaching, %				
Metropolitan nonteaching	44.3	30.0	43.9	26.8
Metropolitan teaching	45.3	60.1	51.8	69.6
Nonmetropolitan	10.4	9.9	4.3	3.6
Length of stay, d	4.0	3.0	4.2	4.3
Index cost, mean, $	16 062	16 520	22 248	26 308

Abbreviation: NA, not applicable.

^a^ The Charlson Comorbidity Index categories are described elsewhere.^19^

### Readmissions

#### Weighted Sample

The weighted unadjusted readmission rates of patients readmitted within 30 days from targeted conditions declined 0.7% from 4.8% in 2010 to 4.1% in 2015 (Table 2). The readmission rates of patients readmitted within 30 days for nontargeted conditions declined 0.1% from 7.4% in 2010 to 7.3% in 2015.

**Table 2.** Weighted Unadjusted Readmission Rates by Procedure, 2010-2015

Procedure	Readmission Rate, %						
	2010	2011	2012	2013	2014	2015	Overall
Targeted	4.8	4.7	4.6	4.3	4.1	4.1	4.4
Total hip arthroplasty	5.2	5.1	4.9	4.6	4.5	4.5	4.8
Total knee arthroplasty	4.7	4.4	4.4	4.1	3.9	3.8	4.2
Nontargeted	7.4	7.3	7.2	7.2	7.1	7.3	7.2
Lumbar spine fusion	7.8	7.7	7.7	7.5	7.4	7.5	7.6
Laminectomy	7.1	7.1	6.9	7.0	7.0	7.1	7.0
Overall	5.6	5.5	5.4	5.1	5.0	4.9	5.2

#### Model

In our model, risk-adjusted rates of readmission during the first period were estimated to decline from 4.60% to 4.12% (slope per month, −0.015%; 95% CI, −0.022% to −0.008%) in the targeted group and from 7.22% to 6.63% (slope per month, −0.018%; 95% CI, −0.030% to −0.007%) in the nontargeted group (Table 3). The readmission rate slopes in the targeted and nontargeted groups during the HRRP preimplementation period were not statistically different (*P* = .07). In the second period, risk-adjusted rates of readmission were estimated to decline from 4.12% to 3.71% (slope per month, −0.018%; 95% CI, −0.025% to −0.010%) in the targeted group and from 6.63% to 6.56% (slope per month, −0.003%; 95% CI, −0.016% to 0.010%) in the nontargeted group. In the HRRP implementation period, the slopes of readmission rates differed in a statistically significant fashion between the targeted and nontargeted group (*P* = .005). Finally, no trends were detected in the HRRP penalty period, for either the targeted or nontargeted group.

**Table 3.** Risk-Adjusted Rates of Readmission in Targeted vs Nontargeted Procedures at Start and End of Given Periods and Slopes^a^

Procedure, Date	Readmission Rate, % (95% CI)	Slope (95% CI)
**Preimplementation**		
Targeted		
January 2010	4.60 (4.43 to 4.78)	−0.015 (−0.022 to −0.008)^b^
October 2012	4.12 (3.99 to 4.24)	
Nontargeted		
January 2010	7.22 (6.93 to 7.52)	−0.018 (−0.030 to −0.007)^b^
October 2012	6.63 (6.43 to 6.84)	
**Implementation**		
Targeted		
October 2012	4.12 (3.99 to 4.24)	−0.018 (−0.025 to −0.010)^b^
October 2014	3.71 (3.60 to 3.83)	
Nontargeted		
October 2012	6.63 (6.43 to 6.84)	−0.003 (−0.016 to −0.010)
October 2014	6.56 (6.35 to 6.77)	
**Penalty**		
Targeted		
October 2014	3.71 (3.60 to 3.83)	0.010 (−0.008 to 0.029)
September 2015	3.83 (3.66 to 4.00)	
Nontargeted		
October 2014	6.56 (6.35 to 6.77)	0.011 (−0.023 to 0.045)
September 2015	6.68 (6.38 to 6.99)	

^a^ Slope is change in risk-adjusted rate from beginning to end of period divided by number of months in between.

^b^ *P* value for testing H_0_ (null hypothesis): slope = 0; 95% CIs do not contain 0, implying *P* < .05.

Across successive study periods, the decreasing slopes of readmission rates were statistically different between period 2 vs period 3 among targeted procedures. Among nontargeted procedures, however, monthly readmission trends were not altered in successive study periods. Risk-adjusted rates of readmission depicting the trends described above for each study period and for targeted and nontargeted procedures are plotted in the Figure. Our findings were similar when studying an exclusively Medicare population (Table 4).

**Figure.**
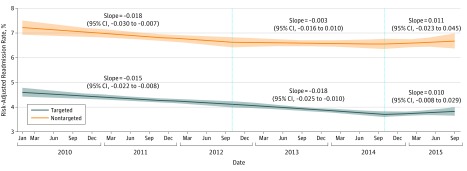
Adjusted Predicted Probability of Readmission Within 30 Days After Discharge for Targeted vs Nontargeted Procedures From 2010 to 2015 Points represent the risk-adjusted predicted probability derived from the linear combination of regression coefficients (converted to the probability scale), set at the mean values of the covariates. Shaded regions indicate the 95% CIs; dashed lines represent divisions between periods.

**Table 4.** Risk-Adjusted Rates of Readmission in Targeted vs Nontargeted Procedures at Start and End of Given Periods and Slopes in Medicare Patients^a^

Procedure, Date	Readmission Rate, % (95% CI)	Slope (95% CI)
**Preimplementation**		
Targeted		
January 2010	5.71 (5.47 to 5.97)	−0.025 (−0.034 to −0.016)^b^
October 2012	4.91 (4.76 to 5.06)	
Nontargeted		
January 2010	8.65 (8.27 to 9.06)	−0.027 (−0.042 to −0.011)^b^
October 2012	7.80 (7.55 to 8.07)	
**Implementation**		
Targeted		
October 2012	4.91 (4.76 to 5.06)	−0.016 (−0.025 to −0.007)^b^
October 2014	4.55 (4.41 to 4.69)	
Nontargeted		
October 2012	7.80 (7.55 to 8.07)	−0.001 (−0.019 to 0.016)
October 2014	7.77 (7.50 to 8.06)	
**Penalty**		
Targeted		
October 2014	4.55 (4.41 to 4.69)	0.006 (−0.017 to 0.029)
September 2015	4.61 (4.41 to 4.82)	
Nontargeted		
October 2014	7.77 (7.50 to 8.06)	0.019 (−0.029 to 0.068)
September 2015	7.99 (7.56 to 8.44)	

^a^ Slope is change in risk adjusted rate from beginning to end of period divided by number of months in between.

^b^ *P* for testing H_0_ (null hypothesis): slope = 0; 95% CI not including 0 implies *P* < .05.

## Discussion

In this nationwide, all-payer, readmissions database, we found that the HRRP was associated with a greater decrease in readmissions after targeted surgical procedures when compared with similar nontargeted procedures. From 2010 to 2015, readmissions declined for all procedures considered. Shortly after enactment of the ACA, the risk-adjusted rate of a patient being readmitted significantly and equivalently decreased for all procedures. This initial decrease in the rates of readmission for nontargeted procedures stabilized with no further reductions. Among targeted procedures, however, the rates of readmission continued to decrease at the same rate during HRRP implementation, subsequently stabilizing with the onset of penalties.

Several important observations can be made. First, large decreases in readmission were observed for all procedures before implementation of the HRRP. It is likely that broader factors, such as enactment of the ACA, explain at least some of the observed reductions in surgical readmissions. Inclusion of the HRRP in the ACA likely created future financial incentives that signaled hospitals to reduce surgical readmissions well in advance of penalties. Second, implementation of the HRRP was associated with decreased rates of readmission after targeted surgical procedures not seen with nontargeted procedures. This finding suggests that hospitals specifically focused efforts to develop policies and programs aiming to decrease readmissions after THA and TKA. This finding may have implications for the future inclusion of other surgical procedures in the HRRP. Third, there was no associated spillover; observed decreases in readmissions after nontargeted procedures can be entirely attributed to the period immediately after passage of the ACA. Fourth, penalty onset did not coincide with further decreases in readmission among targeted procedures. This finding puts forward the possibility that THA and TKA are collectively nearing a point of diminishing returns below which further reduction in readmissions is challenging despite the presence of financial penalties.^22^ This abatement in the extent of reduction of surgical readmissions parallels earlier evidence on the association of the HRRP with medical readmissions.^5,6^

These results present questions about the intended goals and future of the HRRP. In the absence of an experimental study, observed reductions in readmission among targeted procedures associated with implementation of the HRRP may provide evidence of program success. Therefore, the Centers for Medicare & Medicaid Services is not wrong to consider expanding the HRRP to target additional procedures.^23^ However, policy makers should first conduct further studies on baseline trends in readmissions among surgical procedures, refine the penalty formula to address known faults,^24^ and study the HRRP’s association with mortality.^25,26^

This current analysis improves on recent studies evaluating the association of the HRRP with surgical readmissions in several ways. First, to our knowledge, our study is the first to evaluate surgical readmissions after HRRP penalty onset. Prior work was limited to the period before 2015 and, therefore, measures how hospitals responded to the future penalties of the HRRP, not to the HRRP itself. Second, our study uses the NRD, which contains information for patients 50 years or older, therefore providing a more complete look at the association of the HRRP with surgical readmissions in the United States than seen in a study exclusively of patients older than 65 years. This point is particularly salient if one looks at the age distribution of targeted procedures: approximately 43% of index admissions were for patients younger than 65 years. Last, we selected a control cohort of individuals undergoing nontargeted procedures sufficiently similar to the population undergoing THA and TKA. Previous studies examining surgical readmissions approximated secular trends by defining the nontargeted group to include 3 to 5 surgical procedures, including colectomy, abdominal aortic aneurysm repair, and others.^8,9,10^ These procedures were likely chosen because they are common among elderly populations, have a high risk of readmission, and are high cost. We argue that the use of lumbar spine fusion and laminectomy as control procedures provides a better understanding of how the HRRP was associated with THA and TKA readmission trends than do a random selection of common surgical procedures. Evidence of parallel trends between the targeted and nontargeted groups prior to implementation of the HRRP further justifies the use of lumbar spine fusion and laminectomy as controls for THA and TKA.

### Limitations

Our study has limitations. First, we could not capture the outcomes of other readmission reduction efforts occurring concurrently via other initiatives, such as accountable care organizations. Second, while the NRD is ideally suited to study temporal trends before and after implementation of the HRRP, it does not capture readmission trends before the ACA was instituted. However, trends in readmissions before 2010 studied elsewhere showed baseline stable rates prior to the ACA. Third, we could not assess for changing attitudes toward selection of patients for surgery; if frail patients were less likely to receive surgery after implementation of the HRRP, the readmission rate would decline because of patient selection and not owing to readmission reduction efforts. Fourth, because of the cross-sectional nature of the NRD, we cannot identify and follow up patients or hospitals over time. Fifth, the NRD did not permit us to study observation services as a secondary outcome, which could be used to prevent a readmission. Sixth, per the HCUP data use agreement, we excluded low-volume hospitals, which could have resulted in underestimated readmission rates. Seventh, as lumbar spine fusion and laminectomy are performed by both orthopedic and neurologic surgeons, it is possible that the respective specialties experienced differing incentives under the HRRP that could not be captured in this study.

## Conclusions

From our findings, readmissions after all surgical procedures appeared to decline after enactment of the ACA. Our findings suggest that targeted procedures experienced further reduction of readmissions with implementation of the HRRP. However, penalties did not appear to be associated with further decreases in surgical readmissions. Readmission trends appear to be consistent with hospitals responding to the future possibility of penalties by reducing readmissions after THA and TKA.

## Notes

Supplement. **eTable 1.** List of Included *ICD-9* Procedure Codes **eTable 2.** List of Excluded *ICD-9* Procedure Codes **eAppendix.** Regression Model
