# Supplementary material for: Comparison of Hospital Readmission After Total Hip and Total Knee Arthroplasty vs Spinal Surgery After Implementation of the Hospital Readmissions Reduction Program
Source: JAMA Netw Open. 2019 May 31;2(5):e194634. doi: 10.1001/jamanetworkopen.2019.4634 (PMC6547226; doi:10.1001/jamanetworkopen.2019.4634)
Supplement: Supplement. — eTable 1. List of Included ICD-9 Procedure Codes eTable 2. List of Excluded ICD-9 Procedure Codes eAppendix. Regression Model [file jamanetwopen-2-e194634-s001.pdf]

Supplementary Online Content

Ramaswamy A, Marchese M, Cole AP, et al. Comparison of hospital readmission after total hip and total knee arthroplasty vs spinal surgery after implementation of the Hospital Readmissions Reduction Program. *JAMA Netw Open.* 2019;2(5):e194634. doi:10.1001/jamanetworkopen.2019.4634

eTable 1. List of Included *ICD-9* Procedure Codes

eTable 2. List of Excluded *ICD-9* Procedure Codes

eAppendix. Regression Model

This supplementary material has been provided by the authors to give readers additional information about their work.

© 2019 Ramaswamy A et al. *JAMA Network Open.*

eTable 1. List of Included *ICD-9* Procedure Codes

**Procedure**	***ICD-9* Procedure Code**
Total hip arthroplasty	8151
Total knee arthroplasty	8154
Lumbar spine fusion	8106, 8107, 8108, 8136, 8137, 8138
Laminectomy	0302, 0309, 8051

© 2019 Ramaswamy A et al. *JAMA Network Open.*

eTable 2. List of Excluded *ICD-9* Procedure Codes

**Procedure**	***ICD-9* Procedure Code**
Fracture of pelvis and lower limbs	73310, 73314, 73315, 73319, 7338, 73381, 73382, 73395, 73396, 73397, 8080, 8081, 8201, 82011, 82012, 82013, 82019, 82030, 82031, 82032, 821, 8210, 8211
Revision surgery	8153, 8155, 8159, 07, 071, 072, 073, 08, 081, 082, 083, 084
Malignancy	1706, 1707, 1709, 1953, 1955, 1985, 199

© 2019 Ramaswamy A et al. *JAMA Network Open.*

eAppendix. Regression Model

$$\ln\left(\frac{p(y_i)}{1-p(y_i)}\right) = \propto_1 + \beta_{11}x_{11_i} + \beta_{12}x_{12_i} + \beta_{13}x_{13_i} + \propto_2 + \beta_{21}x_{21_i} + \beta_{22}x_{22_i} + \beta_{23}x_{23_i} + \beta_{age} * age_i + \beta_{sex} * sex_i + \beta_{caseload} * caseload_i + \beta_{payor} * payor_i + \beta_{zipinc} * zipinc_i + \beta_{bedsize} * bedsize_i$$

$i = \text{patient i}$
$y = \text{readmit status}$
$\propto_i = \text{intercept; non} - \text{targeted}$
$x_{11} = \text{date in period 1; non} - \text{targeted}$
$x_{12} = \text{date in period 2; non} - \text{targeted}$
$x_{13} = \text{date in period 3; non} - \text{targeted}$
$\propto_2 = \text{intercept; non} - \text{targeted}$
$x_{21} = \text{date in period 1; targeted}$
$x_{22} = \text{date in period 2; targeted}$
$x_{23} = \text{date in period 3; targeted}$

© 2019 Ramaswamy A et al. *JAMA Network Open.*
